# Maternal medication use in pregnancy and offspring ASD risk: A prescription-wide, target-informed study

**DOI:** 10.1192/j.eurpsy.2025.10071

**Published:** 2025-08-19

**Authors:** Nina Zaks, Arad Kodesh, Nicole Zatorski, Yifan Wang, Stephen Z. Levine, Sven Sandin, Abraham Reichenberg, Avner Schlessinger, Magdalena Janecka

**Affiliations:** 1 Department of Child and Adolescent Psychiatry, NYU Grossman School of Medicine, New York, NY, USA; 2 Meuhedet Health Services, Haifa, Israel; 3 Department of Community Mental Health, University of Haifa, Haifa, Israel; 4 Department of Pharmacological Sciences, Icahn School of Medicine at Mount Sinai, New York, NY, USA; 5 Department of Internal Medicine, Duke University; 6 Department of Psychiatry, Icahn School of Medicine at Mount Sinai, New York, NY, USA; 7 School of Public Health, Faculty of Social Welfare and Health Sciences, University of Haifa, Haifa, Israel; 8 Seaver Autism Center for Research and Treatment, Icahn School of Medicine at Mount Sinai, New York, NY, USA; 9 Department of Medical Epidemiology and Biostatistics, Karolinska Institutet, Stockholm, Sweden; 10 Environmental Medicine and Climate Science, Icahn School of Medicine at Mount Sinai, New York, NY, USA; 11 AI Small Molecule Drug Discovery Center, Department of Pharmacological Sciences, Icahn School of Medicine at Mount Sinai, New York, NY, USA; 12 Department of Population Health, New York University Grossman School of Medicine, New York, NY, USA

**Keywords:** autism spectrum disorder (ASD), maternal health, medications, pregnancy, psychiatric epidemiology

## Abstract

**Background:**

Certain prescription drugs used during pregnancy are associated with offspring autism spectrum disorder (ASD). Nonetheless, ASD risk following prenatal exposure to most drugs remains unknown. Furthermore, methodological challenges and ethical concerns hinder the scope for causal inference.

**Methods:**

We used a case-cohort study design of a nationally representative sample from Israel to examine the associations between maternal prescription drug use during pregnancy and offspring ASD. To scrutinize these associations, the analyses were (a) adjusted for indication proxy (level 2 Anatomical Therapeutic Chemical (ATC) codes), (b) repeated using shared pharmacological targets as exposures, and (c) inspected further through target-enrichment analysis.

**Results:**

The sample included 1,400 individuals with and 94,713 without an ASD diagnosis. Among all drugs prescribed during pregnancy, five were statistically significantly associated with increased offspring ASD risk after adjustment for indication proxy (e.g., hazard ratio [95% confidence interval] cyproterone = 2.71 [1.17–6.25] and prednisolone = 2.10 [1.27–3.49]), and two with decreased risk (ferrous sulfate = 0.82 [0.68, 0.99] and lynestrenol = 0.43 [0.2, 0.93]). Further analysis revealed four pharmacological targets shared by these drugs, which were themselves associated with ASD (e.g., neuronal acetylcholine receptor α4β4 = 1.45 [1.05–1.99] and serotonin 2b receptor = 1.31 [1.04–1.61]). Enrichment analysis suggested the association between ASD and medications affecting cholinergic and serotonergic signaling.

**Conclusions:**

Increased ASD risk followed prenatal exposure to five prescription drugs, and decreased risk followed exposure to two. Subsequent analyses suggested no confounding by indication in these associations, but further studies are warranted.

## Introduction

Autism spectrum disorder (ASD) is a neurodevelopmental condition characterized by differences in social communication and restricted, stereotyped behaviors. While the prevalence of ASD has increased from 1 in 150 to 1 in 31 over the past two decades [1, 2], the underlying etiology remains largely unexplained [3]. Maternal prescription drug use in pregnancy has been proposed to be an etiological factor for ASD [4], as evidence suggests that drugs taken during pregnancy could have the potential to both increase [4] and reduce [5, 6] the likelihood of offspring ASD. Nonetheless, at present, ASD risk following prenatal exposure to most prescription drugs remains untested, and the mechanisms underlying the few observed associations remain unexplained.

An improved understanding of prenatal exposure to prescription drugs and their impact on (neuro)development is critical. Over half of pregnant females take a prescribed or over-the-counter medication [7, 8]. This rate is likely to rise, accompanied by the steadily increasing average maternal age at childbirth and the associated burden of chronic maternal conditions in pregnancy [9–11]. Despite this, the studies, to date have primarily focused on the impact of relatively few groups of drugs, such as antiepileptics and antidepressants [12, 13].

For the drugs that have been studied, methodological challenges in the design and analysis of observational studies limit the strength of conclusions. One of the most notable of these limitations is *confounding by indication*, which occurs when the clinical indication for a particular treatment also affects the outcome of interest [14]. For example, if the severity of an illness – justifying the use of a particular drug during pregnancy – also affects the risk of offspring ASD. The role of confounding by maternal indication in observational studies remains unresolved [15]. Namely, it is unclear whether certain maternal conditions (or the upstream familial factors) themselves drive ASD risk, rather than the drugs used to treat them, as deliberated in studies of depression and antidepressant use in pregnancy [16]. This hinders conclusive insights about the causal effects of prenatal exposure to different prescription drugs on neurodevelopment.

Advancing methods to address confounding by indication in observational data is crucial, considering the limitations of clinical trials in pregnancy. Until the 1990s, pregnant females were excluded from trials largely due to ethical and liability considerations [17, 18]. While the more recent trials have allowed their inclusion, those studies still typically lack statistical power to derive adequate inferences about the pregnant population [19–21]. Furthermore, these trials typically do not measure outcomes in the offspring, especially those that, similar to ASD, require a years-long follow-up period. Therefore, our understanding of many of the potential effects of prenatal exposure to prescription drugs relies on accurate inference from observational studies [19], with the existing caveats like confounding by indication.

The current study aims to draw more comprehensive and robust conclusions about the potential role of prenatal exposure to prescription drugs in ASD. In so doing, we (a) harness large-scale, comprehensive electronic health records that include information on indications and prescription records with family linkage; (b) increase the range of prescription drugs and supplements studied in the context of ASD risk; and (c) enhance the scope of causal inference about the potential etiological role of these drugs in ASD by leveraging information on their pharmacological properties and maternal indications. Hence, we integrate approaches from pharmacological sciences, bioinformatics, and epidemiology in electronic health records. Collectively, these approaches may provide novel insights into the range of associations between maternal prescription drug use in pregnancy and offspring ASD, and suggest possible mechanisms underlying these associations.

## Methods

### Sample

We used an established, nationally representative case-cohort sample from the Meuhedet health maintenance organization (HMO) in Israel [4, 6]. The source population includes all children born in Israel from 1998 to 2008 in Meuhedet HMO (*N* = 270,799), and the final sample consists of 35.5% of the source population. See details are provided in Supplementary Materials.

### Exposure

In our prescription-wide approach, we analyzed the exposure to all prescribed drugs with an ATC code and entered in the HMO prescription register, including both medications and supplements prescribed by the HMO health provider. Exposure to each of these drugs was defined as a dichotomized variable indicating maternal prescription (yes/no) of the given drug, identified through its full ATC code in the prescription register. Each ATC code served as a separate medication exposure (irrespective of different trade names under which the chemical formulation was given). The same compounds with multiple ATC codes (e.g., due to different indications) were analyzed as the same medication exposure.

Exposure was ascertained if a mother received a prescription during the 12-month interval (365 days) before childbirth, irrespective of quantity or redemption date (see Supplementary Materials for details).

### Outcome

Individuals with ASD were ascertained using the autism diagnoses in *International Classification of Diseases (ICD) Ninth and Tenth Revisions* (ICD-9: 299 and ICD-10: F84 codes; see Supplementary Table S1 for all subcodes and Supplementary Materials for further detail).

### Covariates

To account for potential temporal trends in ASD diagnosis and prescription drug use, in all models, we adjusted for the child’s birth year. Similarly, maternal age at childbirth has been shown to be associated with maternal and child health outcomes and was hence included in all models. We also adjusted for child sex as it is strongly associated with ASD [22], and for the total number of maternal encounters with health services in the 12 months before childbirth, a proxy for maternal healthcare utilization around pregnancy. The total number of maternal encounters with health services was defined as the number of distinct occasions (based on the date of the encounter) in the exposure period when mothers received any ICD-9 diagnosis.

To address potential confounding by indication, we identified the broad, primary therapeutic use for each drug according to its level 2 ATC codes (e.g., A10 [drugs used in diabetes] for metformin [ATC: A10BA02]). Although broad therapeutic use does not specifically define an indication, it was used as a general proxy in the current study. Henceforth, we refer to it by the term “indication.”

### Statistical analysis: Prescription-wide association study (RxWAS)

In RxWAS analyses, we estimated the associations between each prescription drug exposure and offspring ASD using Cox proportional hazards regression models adjusted for the covariates described above. We examined Schoenfeld residuals to assess the proportional hazards assumption [23]. Due to the presence of siblings in the dataset, all models were adjusted for familial clustering by applying a sandwich estimator and obtaining robust standard errors based on clustering on maternal ID [24] (see Supplementary Materials for details on differential selection probability).

To avoid sparse data bias [25], the primary and secondary analyses (described below) were only performed on exposures with a minimum frequency of five ASD and non-ASD child–mother pairs.

To assess potential confounding by indication, we repeated the RxWAS analyses, adjusting for the indications (broad therapeutic uses/level 2 ATC codes) for each drug. Drugs with only one indication could not be included in these models because their effect estimates were nonidentifiable. To address potential issues related to high collinearity between indication and drug of interest, we estimated the variance inflation factor (VIF) in all models and only included indication-adjusted associations for drugs with a VIF of <5 [26]. Throughout the analyses, we used a two-sided 5% level of significance for hypothesis testing. No multiple testing correction was performed. All regressions were performed using the “survival” package [27]. All analyses were conducted using R software version 4.0.

### Secondary analyses: Mechanisms

**Figure 1.**
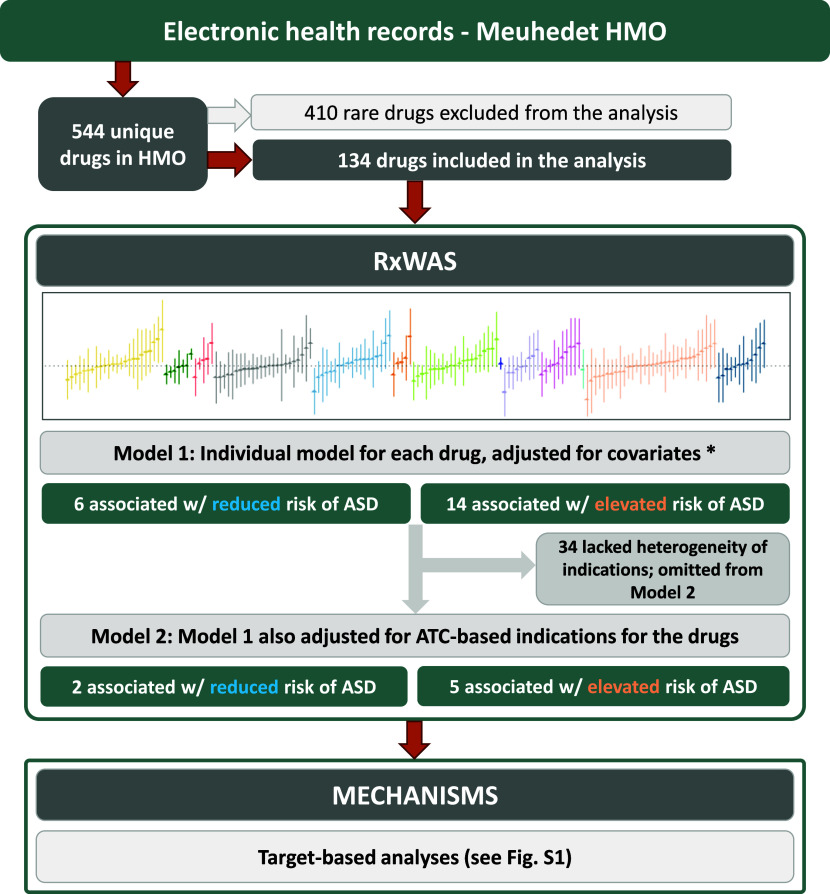
Study overview. *Covariates include maternal age at childbirth, total number of encounters with health care services, and child sex and birth year.

To understand the source of the observed associations and elucidate potential confounding, in secondary analyses we focused on the prescription drugs statistically significantly associated with ASD in the RxWAS (see Figure 1 for the study overview and details below).

### Target-based analysis

We used ChEMBL [28] to perform target annotation of all prescription drugs in our dataset, defining targets as human proteins with which the drug interacts at half-maximal inhibitory concentration (IC_50_) ≤ 1 μM [29]. Target exposures were defined by the protein targets of the RxWAS-significant drugs. Each target exposure included all drugs with this target, including those that were nonsignificant in the RxWAS. The association between each target exposure during the exposure interval and offspring ASD was estimated after adjustment for covariates and all (level 2, ATC-based) indications of the target group, analogously to the main analyses.

Based on the goal of our approach to minimize confounding by indication in the presence of heterogeneity of indications within an exposure, we did not analyze the target exposures where all underlying drugs were prescribed for the same ATC-based indication.

### Enrichment analysis

The targets statistically significantly associated with offspring ASD after controlling for the study covariates and ATC-based indications were further analyzed with Enrichr [30] to determine associated pathways, ontologies, diseases, and cell types. In brief, enrichment analysis is a computational method for inferring knowledge about an input gene or protein by comparing it to those annotated with prior biological knowledge. Enrichment analysis detects whether the input sets of genes/proteins overlap with annotated sets, highlighting statistically significant annotations. Here, all analyzed pharmacological targets were mapped onto genes encoding their protein product, providing the list of input genes for the analyses. We used gene annotations from two representative datasets related to protein function, including WikiPathways [31] and Gene Ontology (GO) Molecular Function [32].

## Results

Our sample included 96,113 children, including 1,400 children with and 94,713 without ASD, born to 35,254 mothers. The mean (standard deviation) age of children at the end of the study follow-up was 11.6 (3.0). Table 1 presents the characteristics of the analytic sample by ASD status.

**Table 1.** Demographic characteristics of the analytic sample, by ASD status

Demographic variable	ASD (*n* = 1,400)	No ASD (*n* = 94,713)
Child’s year of birth		
1998, *n* (%)	72 (5.1%)	7,337 (7.7%)
1999, *n* (%)	106 (7.6%)	8,849 (9.3%)
2000, *n* (%)	120 (8.6%)	8,920 (9.4%)
2001, *n* (%)	129 (9.2%)	8,996 (9.5%)
2002, *n* (%)	131 (9.4%)	9,336 (9.9%)
2003, *n* (%)	173 (12.0%)	9,465 (10.0%)
2004, *n* (%)	171 (12.0%)	9,319 (9.8%)
2005, *n* (%)	153 (11.0%)	9,047 (9.6%)
2006, *n* (%)	163 (12.0%)	8,672 (9.2%)
2007, *n* (%)	148 (11.0%)	8,856 (9.4%)
2008, *n* (%)	34 (2.4%)	5,916 (6.2%)
Child’s sex		
Female, *n* (%)	268 (19.1%)	46,662 (49.3%)
Child’s age at the time of ASD diagnosis (years)		
Median (IQR)	5.9 (3.7, 8.5)	*-*
Maternal age at delivery (years)		
Median (IQR)	29.6 (26.0, 33.7)	29.4 (25.6, 33.5)
Number of drugs prescribed to mother during pregnancy		
Median (IQR)	3.0 (0.0, 6.0)	3.0 (1.0, 6.0)

Abbreviation: IQR, interquartile range.

### Prescription drug exposure, their indications, and pharmacological targets

We identified 908 unique prescription drug names in the dataset, which, after collapsing brand and trade names, mapped onto 544 drugs prescribed to pregnant females in the sample during the exposure period (including medications and supplements, as long as they were prescribed by a healthcare professional). The median number of distinct drugs prescribed to each pregnant female during this period was 3 (interquartile range [IQR]: 1–6), both among mothers of ASD cases and controls.

Most prescription drugs (83%) corresponded to only one ATC-based indication (Supplementary Table S2). Conversely, the median number of unique drugs prescribed for each ATC-based indication was 5 (IQR: 2–10). Of the 544 unique drugs recorded in the dataset, 410 had a frequency of less than five ASD or non-ASD child–mother pairs and, hence, were omitted from the analyses (Figure 1 and Supplementary Table S2).

### RxWAS

**Figure 2.**
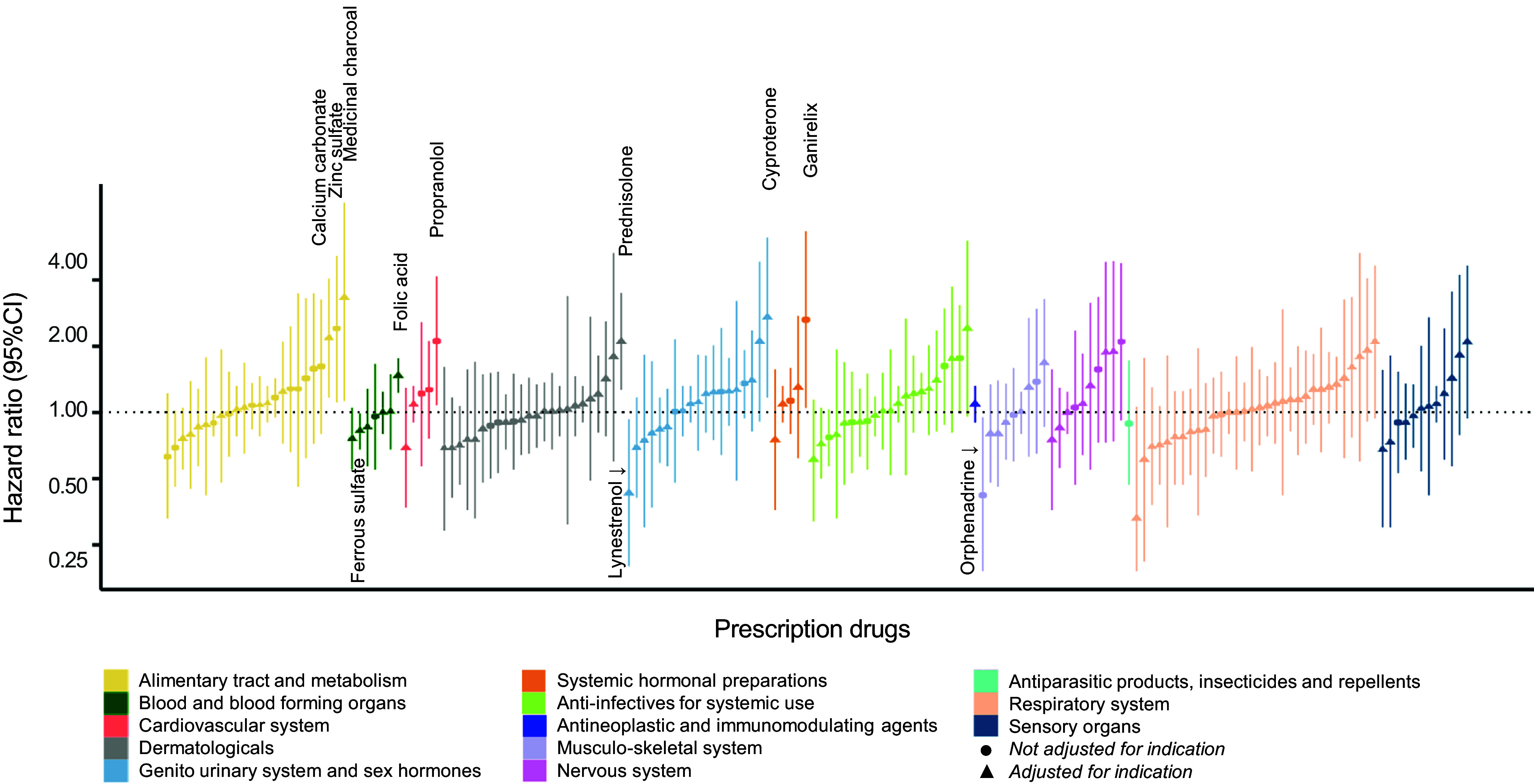
RxWAS: Visual summary of associations between maternal prescription drug exposure and offspring ASD risk. Each bar represents the association between a single drug prescribed in pregnancy and offspring ASD risk (hazard ratios (HR) with 95% confidence intervals (CIs), after covariate adjustment). Bars are grouped and colored by the indication of each drug, defined as its level 2 ATC code (see the legend). The drugs for which analytical adjustment for indication was not possible (due to fully overlapping drug and ATC-based indication category) are indicated with circular, rather than triangular, point estimate markers.

Of the 134 drugs analyzed in our study, 20 were statistically significantly associated with offspring ASD risk after adjustment for covariates. Among those, seven remained statistically significantly associated with offspring ASD after controlling for their ATC-based indications. Four could not be adjusted for indication due to a lack of heterogeneity in indications for those drugs, and while not analyzed as intended, they remain in the study analyses as their potential associations with offspring risk of ASD could not be ruled out. The strongest indication-adjusted associations in the RxWAS analysis included the following: medicinal charcoal (supplement, hazard ratio [HR] = 3.33, 95% confidence interval [CI] = 1.13–9.82), cyproterone (antiandrogen, HR = 2.71, 95% CI = 1.17–6.25), and prednisolone (corticosteroid, HR = 2.10, 95% CI = 1.27–3.49; Figures 1 and 2, Table 2, and Supplementary Table S3).

**Table 2.** RxWAS: Statistical summary of associations between maternal prescription drug exposure and offspring ASD

Prescription drug used in pregnancy	Offspring ASD HR [95% CI]
Calcium carbonate^a^	2.18 [1.17–4.06]
Cyproterone	2.71 [1.17–6.25]
Ferrous sulfate^a^	0.82 [0.68–0.99]
Folic acid^a^	1.47 [1.23–1.76]
Lynestrenol	0.43 [0.2–0.93]
Medicinal charcoal^a^	3.33 [1.13–9.82]
Prednisolone	2.10 [1.27–3.49]
Ganirelix^b^	2.64 [1.05–6.67]
Orphenadrine^b^	0.42 [0.19–0.95]
Propranolol^b^	2.11 [1.08–4.16]

a Prescription supplement, rather than medication.

b Indication adjustment not possible (associations not ruled out).

### Target-based analysis

To identify potential mechanisms through which prescription drugs are associated with offspring ASD, we analyzed the pharmacological targets of the RxWAS-significant drugs. We identified 22 targets that are acted upon by the RxWAS-significant drugs. These protein targets were used to define target exposures, that is, groups of prescription drugs acting on the same pharmacological target as the RxWAS-significant drugs. Four of these target exposures were statistically significantly associated with offspring ASD after controlling for covariates, including ATC-based indications (Table 3 and Supplementary Table S4). We did not replicate the previously reported associations between exposure to medications acting on the serotonin transporter and ASD.

**Table 3.** Associations between pharmacological targets of drugs prescribed in pregnancy and offspring ASD, and frequencies of each target exposure by offspring ASD status

Pharmacologic target	Target analysis HR [95% CI]	Rx in pregnancy associated with offspring ASD	ASD *n* (%)	No ASD *n* (%)
Acetylcholine receptor; alpha1/beta1/delta/gamma	0.96 [0.58–1.59]	*Medicinal charcoal*	21 (1.5%)	1391 (1.5%)
		*Lynestrenol*		
ADAM–17	1.41 [0.91–2.18]	*Prednisolone*	27 (1.9%)	1537 (1.6%)
Beta–2 adrenergic receptor	1.00 [0.62–1.59]	*Propranolol*	45 (3.2%)	2730 (2.9%)
**Butyrylcholinesterase**	**2.04 [1.13–3.69]**	*Cyproterone*	14 (1.0%)	372 (0.4%)
**Carbonic anhydrase IX**	**1.81 [1.05–3.13]**	*Cyproterone*	15 (1.1%)	527 (0.6%)
Cytochrome P450 2D6	1.03 [0.86–1.23]	*Orphenadrine*	546 (39.0%)	38417 (40.6%)
Glucocorticoid receptor	0.99 [0.82–1.20]	*Prednisolone*	514 (36.7%)	37663 (39.8%)
G-protein-coupled bile acid receptor 1	1.41 [0.91–2.18]	*Prednisolone*	27 (1.9%)	1537 (1.6%)
Interleukin–6	1.41 [0.91–2.18]	*Prednisolone*	27 (1.9%)	1537 (1.6%)
Mineralocorticoid receptor	1.04 [0.85–1.28]	*Prednisolone*	496 (35.4%)	35995 (38.0%)
Muscarinic acetylcholine receptor M1	0.97 [0.60–1.56]	*Orphenadrine*	278 (19.9%)	18929 (20.0%)
Muscarinic acetylcholine receptor M2	0.96 [0.58–1.59]	*Orphenadrine*	36 (2.6%)	2199 (2.3%)
Muscarinic acetylcholine receptor M3	0.97 [0.61–1.55]	*Orphenadrine*	281 (20.1%)	19028 (20.1%)
Muscarinic acetylcholine receptor M4	0.96 [0.59–1.54]	*Orphenadrine*	278 (19.9%)	18936 (20.0%)
Muscarinic acetylcholine receptor M5	0.90 [0.57–1.43]	*Orphenadrine*	281 (20.1%)	19089 (20.2%)
**Neuronal acetylcholine receptor; alpha4/beta4**	**1.45 [1.05–1.99]**	*Cyproterone*	53 (3.8%)	2178 (2.3%)
Peptidyl-prolyl cis-trans isomerase NIMA-interacting 1	0.86 [0.53–1.42]	*Lynestrenol*	20 (1.4%)	1515 (1.6%)
		*Cyproterone*		
Serotonin 2a (5-HT2a) receptor	0.96 [0.66–1.39]	*Orphenadrine*	293 (20.9%)	19724 (20.8%)
**Serotonin 2b (5-HT2b) receptor**	**1.31 [1.04–1.65]**	*Propranolol*	400 (28.6%)	25443 (26.9%)
Serotonin 2c (5-HT2c) receptor	1.09 [0.83–1.45]	*Orphenadrine*	331 (23.6%)	22090 (23.3%)
Serotonin 3a (5-HT3a) receptor	1.13 [0.72–1.77]	*Calcium carbonate*	26 (1.9%)	1716 (1.8%)
		*Medicinal charcoal*		
		*Lynestrenol*		
Serotonin transporter	0.99 [0.80–1.23]	*Propranolol*	201 (14.4%)	13334 (14.1%)
		*Orphenadrine*		

*Note:* Rx = prescription. The included targets are those of the RxWAS-significant drugs. The target exposures are groupings of prescription drugs with the same pharmacologic targets as the RxWAS-significant drugs, irrespective of their significance in the RxWAS. HRs are presented after adjustment for maternal age at delivery, number of encounters with healthcare services, child sex and year of birth, and ATC-based indications. Associations that are nominally significant.

Pharmacological targets significantly associated with offspring ASD included butyrylcholinesterase (HR = 2.04, 95% CI = 1.13–3.69), carbonic anhydrase IX (HR = 1.81, 95% CI = 1.05–3.13), neuronal acetylcholine receptor, alpha4/beta4 (HR = 1.45, 95% CI = 1.05–1.99), and serotonin-2b receptor (HR = 1.31, 95% CI = 1.04–1.65).

### Enrichment analysis

**Figure 3.**
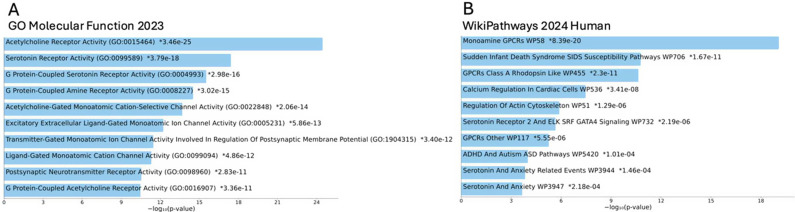
Enrichment analysis of the 22 targets of ASD-associated prescription drugs. (A) Enrichment analysis using the Gene Ontology (GO) Molecular Function 2023 gene-set library. (B) Enrichment analysis using the WikiPathways 2024 Human gene-set library. Enriched terms are ranked according to -log10(p-value), and p-value is calculated using Fisher’s exact test. The bar charts display the top 10 ranked molecular functions and biological pathways.

To identify biological mechanisms associated with the significant protein targets, we performed enrichment analysis to determine pathways, ontologies, diseases, and cell types associated with the target exposures. Applying Enrichr to the significant targets revealed a variety of protein characteristics of drugs associated with offspring ASD (Figure 3 and Supplementary Figure S1). The analysis of the GO framework 2024 (Figure 3A) highlights the role of serotonergic and acetylcholine receptors, as well as other ion channels and neurotransmitter functions. Pathway analysis using WikiPathways corroborates these findings, revealing broader mechanisms such as monoamine G protein-coupled receptor (GPCR) pathways as well as attention-deficit/hyperactivity disorder (ADHD)- and ASD-related pathways (Figure 3B).

## Discussion

Using a nationally representative case-cohort sample, we systematically evaluated the associations between maternal exposure to prescription drugs during pregnancy and offspring ASD, while examining the presence of confounding by indication in these associations. To this end, we performed an RxWAS supported by (a) adjustment for the heterogeneous therapeutic uses of those drugs; (b) the creation of new exposure categories based on the pharmacological targets of the drugs associated with offspring ASD in the RxWAS; and (c) enrichment analysis of those pharmacological targets.

Implementing a prescription-wide approach enabled us to examine the effects of a wide range of maternal medications and supplements on offspring ASD. The follow-up time of the included children ranged from 6 to 17 years, mitigating the potential of missed ASD diagnoses (most children are diagnosed by the age of 5 years [33]). Among the drugs we tested, 11 were nominally associated with ASD, and for 7 of those we validated that the associations persisted after adjusting for the relevant ATC level 2 therapeutic/pharmacological subgroup, which was used as a broad proxy for indication in our study. These included prednisolone (a corticosteroid), cyproterone (an antiandrogen), and lynestrenol (a progestogen), as well as prescribed food supplements, including calcium carbonate, folic acid, ferrous sulfate, and medicinal charcoal.

While our approach demonstrates the utility of an agnostic, prescription-wide approach to identify novel associations and formulate new hypotheses regarding the impact of prescription drug exposure on neurodevelopment, we emphasize that the associations reported in the study require further replication and validation in independent samples. For example, while the associations with the prescribed food supplements were nominally significant and robust to adjustment for ATC-based indication proxy, we cannot exclude a potential role of ascertainment bias in these results. As these supplements are readily available over the counter, prescription records may indicate pregnancies with more severe underlying health problems (e.g., vitamin deficiencies during pregnancy have been linked to higher rates of offspring ASD [6, 34–36]). Importantly, the protective effect of folic acid on fetal neurodevelopment has been extensively demonstrated [37], and folic acid remains a critical supplement before and during pregnancy [38].

As an orthogonal approach to further examine RxWAS associations, we grouped drugs by their pharmacological targets to create alternative exposures. This method offered statistical power to elucidate functional signals of the aggregated effects of multiple drugs that act upon the same pharmacological target, but were prescribed for heterogeneous conditions. In these analyses, with adjustment for all ATC-based indication proxies, we observed that four pharmacological targets of drugs prescribed in pregnancy – butyrylcholinesterase, carbonic anhydrase IX, neuronal acetylcholine receptor α4β4, and serotonin 2b (5-HT2b) receptor – were associated with offspring ASD. None of these pharmacological targets has an established link to ASD etiology, and most of the included drugs do not act upon those targets directly but interact secondarily, warranting further exploration into these targets with respect to neurodevelopment. Nonetheless, the involvements of cholinergic and serotonergic transmission were also highlighted in the additional enrichment analysis, underscoring the potential role of acetylcholine and serotonin in the etiology of ASD. The enrichment analysis provided further validation that the targets identified in our study are relevant to central nervous system signaling, autism, and ADHD (which is frequently comorbid, shares clinical features, and has etiological overlap with ASD).

In line with existing findings [7, 8], our study underscores the frequency of prenatal exposure to prescription drugs and, therefore, the importance of studying medications and supplements on diverse (neuro)developmental outcomes. We reaffirm that confounding by maternal indication in pharmacoepidemiologic research is pervasive, and encourage the development of accurate tools to minimize its impact. Elucidating the potentially deleterious effects of prescription drugs on child health has important implications for disease management during pregnancy; at the same time, nonadherence to certain prescription drugs remains a challenge among pregnant women [39] and can be harmful to both the mother and the fetus. Therefore, considering that a number of indications themselves are associated with ASD risk, it is equally important to highlight the prescription drugs *not* associated with the risk of adverse child outcomes to reassure pregnant females and improve adherence.

### Strengths and limitations

Our results provide novel insights into potentially modifiable factors for ASD, enhance current methods of exploring important influences of neurodevelopment, and suggest that prescription medications and supplements warrant further interrogation in the context of ASD. Based on orthogonal approaches from pharmacological sciences, bioinformatics, and epidemiology in our design and analyses, we implemented a method that explicitly recognizes and capitalizes on the pharmacological characteristics of prescription drugs to reduce confounding by indication and offer mechanistic insights. Although our study focused on ASD, the methodology and its implications are pertinent to understanding and minimizing confounding by indication when analyzing other outcomes.

Our study has several limitations. Given the lack of complete data on drug redemption, we defined the exposure based on prescription data, which could result in potential misclassification of exposure (e.g., in instances of nonadherence to the treatment regimen). Nevertheless, considering the universal health coverage and prescription affordability in Israel, we do not expect a differential misclassification/nonadherence with respect to socioeconomic characteristics or the outcome. Additionally, we did not account for the rare instances in which individuals left Meuhedet HMO, potentially resulting in misclassification of age at censoring. Furthermore, adjustment for indication was based on an indication proxy (i.e., level 2 ATC codes), which does not offer detailed clinical information, potentially affecting the effectiveness of this adjustment. Next, even though the sample size was relatively large, the study remained underpowered to examine the rare prescription drugs and for evaluating exposures by timing, dose, frequency, duration, mode of administration, and – often – maternal indication.

The potential for type I error across analyses must also be considered, as well as the need to replicate our results in independent datasets. Additionally, since drug exposures can co-occur due to polypharmacy, that is, the concurrent use of multiple medications, and each of these drugs may have multiple pharmacological targets, some of the associations may not reflect true causal relationships. Instead, they could represent false-positive results arising from correlations between exposures in our study. Our decision not to concurrently adjust for multiple drugs/targets was dictated by a lack of a strong hypothesis about the causal relationships between those exposures, and in the RxWAS, focus on associations that are amenable to subsequent mechanistic follow-up.

Moreover, there is no information regarding the dosage of the prescribed drugs, and the cutoffs used to define the target are permissive, which may not accurately reflect their concentrations in patients. This issue is even more pronounced with dietary supplements, which are unregulated both in terms of dosage and pharmacological profiling. Therefore, any analysis involving these supplements should be approached with particular caution. Lastly, while our approach provided an improved control for confounding by indication, it did not eliminate all confounding factors, including familial and other residual confounding. Critically, the nature of our analyses remains exploratory. While the observed associations provide signals about the potential effects of maternal use of prescription drugs on offspring ASD risk, drawing further causal conclusions will require validation of these findings in experimental, hypothesis-driven studies.

## Conclusion

Driven by a multidisciplinary approach, findings from a nationally representative birth cohort showed several associations between maternal use of certain prescription drugs in pregnancy and offspring ASD risk. In follow-up analyses, preliminary results showed a limited role of confounding by indication in these associations, but replication and validation of these effects is required. Our approach provides a novel framework for detecting links between maternal prescription drug use in pregnancy and offspring ASD risk while disentangling the underlying mechanisms.

## Supporting information

10.1192/j.eurpsy.2025.10071.sm001 Zaks et al. supplementary material Zaks et al. supplementary material

## Data Availability

Due to data security, privacy considerations, and our agreement with the data provider, Meuhedet, we are unable to share individual-level HMO data. Verified researchers may request access to the data directly from Meuhedet, subject to review and approval of their request. Our request was processed by the study author Levine.

## Supplementary material

The supplementary material for this article can be found at http://doi.org/10.1192/j.eurpsy.2025.10071.
