# Supplementary material for: Maternal medication use in pregnancy and offspring ASD risk: A prescription-wide, target-informed study
Source: Eur Psychiatry. 2025 Aug 19;68(1):e125. doi: 10.1192/j.eurpsy.2025.10071 (PMC12438979; doi:10.1192/j.eurpsy.2025.10071)
Supplement: Zaks et al. supplementary material [file S0924933825100710sup001.docx]

**Maternal medication use in pregnancy and offspring ASD risk: a prescription-wide, target-informed study**

**Supplemental materials**

Contents

[**Supplementary Methods** 2](#_Toc199852999)

[**Table S1.** ICD-9 and ICD-10 codes used to identify ASD cases. 4](#_Toc199853000)

[**Table S2**. Exposure frequencies to the 134 most common drugs prescribed to mothers during pregnancy (i) by offspring ASD status and (ii) stratified by ATC-based indication. 5](#_Toc199853001)

[**Table S3.** Prescription-wide (RxWAS) associations between maternal prescription drug exposure during pregnancy and risk of offspring ASD before and after adjustment for ATC-based indications. 12](#_Toc199853002)

[**Table S4.** Associations between maternal prescription drug exposure and risk of offspring ASD by pharmacologic target. 16](#_Toc199853003)

[**Figure S1.** Target-based analysis flow. 20](#_Toc199853004)

# **Supplementary Methods**

Sample (complete description)

We used an established nationally representative case-cohort sample from the Meuhedet health maintenance organization (HMO) in Israel [1, 2]. All citizens in Israel are required to choose medical insurance from four equivalent plans from four different HMOs. These HMOs by law are not permitted to refuse citizens membership on the grounds of geographic location, demographics, medication needs, or diagnostic history. These features limit the risks of selection and ascertainment bias in our sample. We have demonstrated the representativeness of our data, with the age-specific ASD prevalence in the HMO similar to the rates reported in the Israeli population [3], and rates of medication use comparable with those reported in other national registers [1]. The Institutional Review Board of the University of Haifa and the Helsinki Ethics Committee at Meuhedet approved the study and waived the need for informed consent as the data did not include any individually identifiable information.

The source population included all children born in Israel from 1998 to 2008 in Meuhedet HMO (N=270,799). At the initial stage of sampling, a sub-cohort of 19.5% of the source population was selected at random. Additionally, all children with an ASD diagnosis within the source population were also selected into the cohort. Next, all siblings of the sub-cohort and ASD cases within the source population were also included in the sample. The probability sampling resulted in an overlap between the individuals in the sub-cohort and the census of ASD cases; however, each selected individual was only included once in the study sample. The final sample included 96,113 children, or 35.5% of the source population.

Exposure (complete description on maternal exposure window)

The maternal exposure window was the 12-month interval (365 days) before childbirth. This covered the estimated nine months of full-term pregnancy and the three months beforehand, to capture potential carry-over effects of recently prescribed drugs into pregnancy. Exposure was ascertained if a mother received a prescription during this period, irrespective of quantity or redemption date.

Outcome (complete description)

Individuals with ASD were ascertained according to the *International Classification of Diseases (ICD) Ninth and Tenth Revisions* (ICD-9: 299 and ICD-10: F84; see **Table S1** for all subcodes). All suspected cases of ASD were evaluated by an expert panel including social workers, a psychologist, and one of the following: a developmental-behavioral pediatrician, a child neurologist, or a psychiatrist, with the final diagnosis being made by a board-certified developmental-behavioral pediatrician. All children were followed up until January 26, 2015, first ASD diagnosis, or death, whichever occurred first.

Statistical Analysis – Prescription-Wide Association Study (RxWAS) (differential selection probability)

Individuals with ASD and their siblings were all included in the sample, together with a random sample of the source population and their siblings. Therefore, children with more siblings, those with an ASD diagnosis, and those with an affected sibling were more likely to be included in the sample. To account for this differential selection probability of individuals in the analytic sample by ASD diagnosis and number of siblings in the source population, we applied stabilized inverse probability selection weights to all the models [1, 4]. Additionally, to avoid sparse data bias [5], analyses were performed only on the drugs to which a minimum of five ASD and non-ASD child-mother pairs were exposed.

# **Table S1.** ICD-9 and ICD-10 codes used to identify ASD cases.

| **ICD-9** | **ICD-10** |
| --- | --- |
| 299.0: Infantile Autism | F84.0: Autistic disorder |
| 299.1: Childhood disintegrative Disorder | F84.1: Atypical Autism |
| 299.8: Other Pervasive Developmental Disorders | F84.2: Rett Syndrome |
| 299.9: Other Pervasive Developmental Disorders NOS | F84.5: Asperger Syndrome |
|  | F84.8: Other Pervasive Developmental Disorders |
|  | F84.9 Other Pervasive Developmental Disorders NOS |

# **Table S2**. Exposure frequencies to the 134 most common drugs prescribed to mothers during pregnancy (i) by offspring ASD status and (ii) stratified by ATC-based indication.

| **Prescription drug name** | **ASD *n=1,400  (100%)*** | | **No ASD *n=94,713  (100%)*** | **ATC level 2 code** | **ATC-based indication** | **ASD** | | **No ASD** |
| --- | --- | --- | --- | --- | --- | --- | --- | --- |
|  |  |  |  |  |  | **n (%) by indication** | | |
| **Acetylcysteine** | 14 (1.0%) | 720 (0.8%) | | R05 | Cough | 14 (100.0%) | 720 (100.0%) | |
| **Acetylsalicylic acid** | 254 (18.1%) | 17508 (18.5%) | | N02 | Analgesics | 160 (46.9%) | 11385 (51.0%) | |
|  |  |  |  | R05 | Cough | 43 (12.6%) | 1951 (8.7%) | |
|  |  |  |  | R01 | Nasal preparations | 41 (12.0%) | 2446 (11.0%) | |
|  |  |  |  | B01 | Antithrombotic agents | 34 (10.0%) | 2677 (12.0%) | |
|  |  |  |  | M01 | Antiinflammatory | 28 (8.2%) | 1978 (8.9%) | |
|  |  |  |  | R03 | Drugs for obstructive airway diseases | 16 (4.7%) | 769 (3.4%) | |
|  |  |  |  | R06 | Antihistamines for systemic use | 15 (4.4%) | 854 (3.8%) | |
|  |  |  |  | N06 | Psychoanaleptics | 4 (1.2%) | 270 (1.2%) | |
| **Aciclovir** | 15 (1.1%) | 1159 (1.2%) | | D06 | Antibiotics | 14 (87.5%) | 1099 (83.4%) | |
|  |  |  |  | J05 | Antivirals for systemic use | 1 (6.2%) | 182 (13.8%) | |
|  |  |  |  | S01 | Ophthalmologicals | 1 (6.2%) | 37 (2.8%) | |
| **Alprazolam** | 6 (0.4%) | 177 (0.2%) | | N05 | Psycholeptics | 6 (100.0%) | 177 (100.0%) | |
| **Amitriptyline** | 7 (0.5%) | 138 (0.1%) | | N06 | Psychoanaleptics | 7 (100.0%) | 138 (100.0%) | |
| **Amoxicillin** | 272 (19.4%) | 22064 (23.3%) | | J01 | Antibacterials for systemic use | 272 (100.0%) | 22064 (100.0%) | |
| **Anti-d (rh) immunoglobulin** | 54 (3.9%) | 4176 (4.4%) | | J06 | Immune sera | 54 (100.0%) | 4176 (100.0%) | |
| **Artificial tears** | 6 (0.4%) | 693 (0.7%) | | S01 | Ophthalmologicals | 6 (100.0%) | 693 (100.0%) | |
| **Azelastine** | 6 (0.4%) | 223 (0.2%) | | R01 | Nasal preparations | 6 (100.0%) | 223 (100.0%) | |
| **Azithromycin** | 10 (0.7%) | 1010 (1.1%) | | J01 | Antibacterials for systemic use | 10 (100.0%) | 1010 (100.0%) | |
| **Bifonazole** | 66 (4.7%) | 5383 (5.7%) | | D01 | Antifungals for dermatological use | 66 (100.0%) | 5383 (100.0%) | |
| **Bismuth preparations** | 10 (0.7%) | 619 (0.7%) | | A07 | Antidiarrheals | 9 (90.0%) | 521 (83.0%) | |
|  |  |  |  | A02 | Drugs for acid related disorders | 1 (10.0%) | 107 (17.0%) | |
| **Bromhexine** | 24 (1.7%) | 1723 (1.8%) | | R05 | Cough | 24 (100.0%) | 1723 (100.0%) | |
| **Bromocriptine** | 9 (0.6%) | 408 (0.4%) | | G02 | Other gynecologicals | 9 (100.0%) | 408 (100.0%) | |
| **Budesonide** | 15 (1.1%) | 1156 (1.2%) | | R03 | Drugs for obstructive airway diseases | 15 (100.0%) | 1156 (100.0%) | |
| **Cabergoline** | 7 (0.5%) | 380 (0.4%) | | G02 | Other gynecologicals | 7 (100.0%) | 380 (100.0%) | |
| **Calcium carbonate** | 14 (1.0%) | 402 (0.4%) | | A02 | Drugs for acid related disorders | 14 (100.0%) | 402 (100.0%) | |
| **Carbamide** | 9 (0.6%) | 931 (1.0%) | | D02 | Emollients | 9 (100.0%) | 931 (100.0%) | |
| **Carbocisteine** | 47 (3.4%) | 3222 (3.4%) | | R05 | Cough | 47 (100.0%) | 3222 (100.0%) | |
| **Cefalexin** | 36 (2.6%) | 3797 (4.0%) | | J01 | Antibacterials for systemic use | 36 (100.0%) | 3797 (100.0%) | |
| **Cefuroxime** | 159 (11.4%) | 10815 (11.4%) | | J01 | Antibacterials for systemic use | 159 (100.0%) | 10815 (100.0%) | |
| **Celecoxib** | 8 (0.6%) | 372 (0.4%) | | M01 | Antiinflammatory | 8 (100.0%) | 372 (100.0%) | |
| **Cetirizine** | 10 (0.7%) | 435 (0.5%) | | R06 | Antihistamines for systemic use | 10 (100.0%) | 435 (100.0%) | |
| **Chlorphenamine** | 14 (1.0%) | 1254 (1.3%) | | R06 | Antihistamines for systemic use | 14 (100.0%) | 1254 (100.0%) | |
| **Choriogonadotropin alfa** | 5 (0.4%) | 354 (0.4%) | | G03 | Sex hormones | 5 (100.0%) | 354 (100.0%) | |
| **Citalopram** | 7 (0.5%) | 163 (0.2%) | | N06 | Psychoanaleptics | 7 (100.0%) | 163 (100.0%) | |
| **Clarithromycin** | 5 (0.4%) | 413 (0.4%) | | J01 | Antibacterials for systemic use | 5 (100.0%) | 413 (100.0%) | |
| **Clindamycin** | 27 (1.9%) | 1267 (1.3%) | | G01 | Gynecological antiinfectives | 18 (66.7%) | 795 (62.1%) | |
|  |  |  |  | D10 | Anti-acne preparations | 8 (29.6%) | 418 (32.6%) | |
|  |  |  |  | J01 | Antibacterials for systemic use | 1 (3.7%) | 68 (5.3%) | |
| **Clobetasol** | 13 (0.9%) | 1151 (1.2%) | | D07 | Corticosteroids | 13 (100.0%) | 1151 (100.0%) | |
| **Clomifene** | 50 (3.6%) | 2828 (3.0%) | | G03 | Sex hormones | 50 (100.0%) | 2828 (100.0%) | |
| **Combinations** | 54 (3.9%) | 3514 (3.7%) | | A02 | Drugs for acid related disorders | 45 (80.4%) | 3057 (86.2%) | |
|  |  |  |  | G03 | Sex hormones | 6 (10.7%) | 297 (8.4%) | |
|  |  |  |  | B05 | Blood substitutes | 5 (8.9%) | 193 (5.4%) | |
| **Cromoglicic acid** | 6 (0.4%) | 675 (0.7%) | | S01 | Ophthalmologicals | 6 (100.0%) | 675 (100.0%) | |
| **Cyanocobalamin** | 27 (1.9%) | 2019 (2.1%) | | B03 | Antianemic preparations | 27 (100.0%) | 2019 (100.0%) | |
| **Cyproterone** | 6 (0.4%) | 143 (0.2%) | | G03 | Sex hormones | 6 (100.0%) | 143 (100.0%) | |
| **Desloratadine** | 8 (0.6%) | 269 (0.3%) | | R06 | Antihistamines for systemic use | 8 (100.0%) | 269 (100.0%) | |
| **Dexamethasone** | 342 (24.4%) | 25336 (26.8%) | | D01 | Antifungals for dermatological use | 190 (25.4%) | 13130 (24.0%) | |
|  |  |  |  | D07 | Corticosteroids | 136 (18.2%) | 12215 (22.3%) | |
|  |  |  |  | D06 | Antibiotics | 132 (17.6%) | 9252 (16.9%) | |
|  |  |  |  | D10 | Anti-acne preparations | 91 (12.1%) | 5556 (10.2%) | |
|  |  |  |  | G01 | Gynecological antiinfectives | 83 (11.1%) | 5701 (10.4%) | |
|  |  |  |  | D04 | Antipruritics | 36 (4.8%) | 3141 (5.7%) | |
|  |  |  |  | C05 | Vasoprotectives | 29 (3.9%) | 2072 (3.8%) | |
|  |  |  |  | H02 | Corticosteroids for systemic use | 14 (1.9%) | 960 (1.8%) | |
|  |  |  |  | D02 | Emollients | 12 (1.6%) | 593 (1.1%) | |
|  |  |  |  | S01 | Ophthalmologicals | 9 (1.2%) | 749 (1.4%) | |
|  |  |  |  | S02 | Otologicals | 9 (1.2%) | 749 (1.4%) | |
|  |  |  |  | L01 | Antineoplastic agents | 6 (0.8%) | 249 (0.5%) | |
|  |  |  |  | A07 | Antidiarrheals | 2 (0.3%) | 335 (0.6%) | |
|  |  |  |  | D05 | Antipsoriatics | 0 (0%) | 35 (0.1%) | |
|  |  |  |  | J01 | Antibacterials for systemic use | 0 (0%) | 1 (0.0%) | |
| **Dexpanthenol** | 11 (0.8%) | 647 (0.7%) | | D03 | Preparations for treatment of wounds | 7 (50.0%) | 428 (63.5%) | |
|  |  |  |  | D08 | Antiseptics | 6 (42.9%) | 232 (34.4%) | |
|  |  |  |  | R02 | Throat preparations | 1 (7.1%) | 14 (2.1%) | |
| **Diazepam** | 6 (0.4%) | 365 (0.4%) | | N05 | Psycholeptics | 6 (100.0%) | 365 (100.0%) | |
| **Diclofenac** | 60 (4.3%) | 4736 (5.0%) | | M02 | Topical products for joint | 39 (61.9%) | 3262 (63.6%) | |
|  |  |  |  | M01 | Antiinflammatory | 24 (38.1%) | 1850 (36.1%) | |
|  |  |  |  | S01 | Ophthalmologicals | 0 (0%) | 13 (0.3%) | |
| **Doxycycline** | 19 (1.4%) | 1000 (1.1%) | | J01 | Antibacterials for systemic use | 19 (100.0%) | 1000 (100.0%) | |
| **Drospirenone** | 7 (0.5%) | 304 (0.3%) | | G03 | Sex hormones | 7 (100.0%) | 304 (100.0%) | |
| **Enoxaparin** | 15 (1.1%) | 1229 (1.3%) | | B01 | Antithrombotic agents | 15 (100.0%) | 1229 (100.0%) | |
| **Erythromycin** | 33 (2.4%) | 2393 (2.5%) | | D10 | Anti-acne preparations | 27 (81.8%) | 1153 (47.5%) | |
|  |  |  |  | J01 | Antibacterials for systemic use | 6 (18.2%) | 1272 (52.5%) | |
| **Estradiol** | 18 (1.3%) | 1042 (1.1%) | | G03 | Sex hormones | 18 (100.0%) | 1042 (100.0%) | |
| **Etodolac** | 17 (1.2%) | 1291 (1.4%) | | M01 | Antiinflammatory | 17 (100.0%) | 1291 (100.0%) | |
| **Famotidine** | 26 (1.9%) | 1192 (1.3%) | | A02 | Drugs for acid related disorders | 26 (100.0%) | 1192 (100.0%) | |
| **Ferrous sulfate** | 389 (27.8%) | 30637 (32.3%) | | B03 | Antianemic preparations | 389 (100.0%) | 30637 (100.0%) | |
| **Fexofenadine** | 21 (1.5%) | 1285 (1.4%) | | R06 | Antihistamines for systemic use | 21 (100.0%) | 1285 (100.0%) | |
| **Fluconazole** | 13 (0.9%) | 420 (0.4%) | | J02 | Antimycotics for systemic use | 13 (100.0%) | 420 (100.0%) | |
| **Fluoxetine** | 6 (0.4%) | 118 (0.1%) | | N06 | Psychoanaleptics | 6 (100.0%) | 118 (100.0%) | |
| **Fluticasone** | 59 (4.2%) | 3423 (3.6%) | | R01 | Nasal preparations | 54 (90.0%) | 2984 (83.7%) | |
|  |  |  |  | R03 | Drugs for obstructive airway diseases | 6 (10.0%) | 583 (16.3%) | |
| **Folic acid** | 388 (27.7%) | 22484 (23.7%) | | B03 | Antianemic preparations | 388 (100.0%) | 22484 (100.0%) | |
| **Fosfomycin** | 10 (0.7%) | 658 (0.7%) | | J01 | Antibacterials for systemic use | 10 (100.0%) | 658 (100.0%) | |
| **Fusidic acid** | 5 (0.4%) | 298 (0.3%) | | S01 | Ophthalmologicals | 5 (100.0%) | 298 (100.0%) | |
| **Ganirelix** | 5 (0.4%) | 86 (0.1%) | | H01 | Pituitary | 5 (100.0%) | 86 (100.0%) | |
| **Gentamicin** | 10 (0.7%) | 886 (0.9%) | | S01 | Ophthalmologicals | 10 (100.0%) | 886 (100.0%) | |
| **Gestodene** | 18 (1.3%) | 1055 (1.1%) | | G03 | Sex hormones | 18 (100.0%) | 1055 (100.0%) | |
| **Glycerol** | 9 (0.6%) | 846 (0.9%) | | A06 | Drugs for constipation | 9 (100.0%) | 846 (100.0%) | |
| **Hepatitis B** | 11 (0.8%) | 413 (0.4%) | | J07 | Vaccines | 11 (100.0%) | 413 (100.0%) | |
| **Ipratropium bromide** | 5 (0.4%) | 479 (0.5%) | | R03 | Drugs for obstructive airway diseases | 5 (100.0%) | 479 (100.0%) | |
| **Isoconazole** | 46 (3.3%) | 4905 (5.2%) | | D07 | Corticosteroids | 43 (51.8%) | 4673 (53.7%) | |
|  |  |  |  | D01 | Antifungals for dermatological use | 40 (48.2%) | 4032 (46.3%) | |
| **Ispaghula** | 6 (0.4%) | 323 (0.3%) | | A06 | Drugs for constipation | 6 (100.0%) | 323 (100.0%) | |
| **Ispaghula (psylla seeds)** | 5 (0.4%) | 314 (0.3%) | | A06 | Drugs for constipation | 5 (100.0%) | 314 (100.0%) | |
| **Ketoconazole** | 16 (1.1%) | 1062 (1.1%) | | D01 | Antifungals for dermatological use | 15 (93.8%) | 941 (86.9%) | |
|  |  |  |  | J02 | Antimycotics for systemic use | 1 (6.2%) | 142 (13.1%) | |
| **Lactulose** | 11 (0.8%) | 557 (0.6%) | | A06 | Drugs for constipation | 11 (100.0%) | 557 (100.0%) | |
| **Levocabastine** | 8 (0.6%) | 373 (0.4%) | | S01 | Ophthalmologicals | 7 (87.5%) | 317 (81.5%) | |
|  |  |  |  | R01 | Nasal preparations | 1 (12.5%) | 72 (18.5%) | |
| **Levothyroxine sodium** | 41 (2.9%) | 2567 (2.7%) | | H03 | Thyroid therapy | 41 (100.0%) | 2567 (100.0%) | |
| **Lidocaine** | 9 (0.6%) | 764 (0.8%) | | D04 | Antipruritics | 7 (70.0%) | 627 (77.2%) | |
|  |  |  |  | H02 | Corticosteroids for systemic use | 2 (20.0%) | 134 (16.5%) | |
|  |  |  |  | N01 | Anesthetics | 1 (10.0%) | 51 (6.3%) | |
| **Loperamide** | 16 (1.1%) | 1105 (1.2%) | | A07 | Antidiarrheals | 16 (100.0%) | 1105 (100.0%) | |
| **Loratadine** | 55 (3.9%) | 2836 (3.0%) | | R06 | Antihistamines for systemic use | 55 (100.0%) | 2836 (100.0%) | |
| **Lorazepam** | 7 (0.5%) | 319 (0.3%) | | N05 | Psycholeptics | 7 (100.0%) | 319 (100.0%) | |
| **Lynestrenol** | 7 (0.5%) | 1014 (1.1%) | | G03 | Sex hormones | 7 (100.0%) | 1014 (100.0%) | |
| **Mebendazole** | 11 (0.8%) | 1175 (1.2%) | | P02 | Anthelmintics | 11 (100.0%) | 1175 (100.0%) | |
| **Medicinal charcoal** | 5 (0.4%) | 126 (0.1%) | | A07 | Antidiarrheals | 5 (100.0%) | 126 (100.0%) | |
| **Metamizole sodium** | 30 (2.1%) | 2719 (2.9%) | | N02 | Analgesics | 30 (100.0%) | 2719 (100.0%) | |
| **Metformin** | 7 (0.5%) | 249 (0.3%) | | A10 | Drugs used in diabetes | 7 (100.0%) | 249 (100.0%) | |
| **Methyldopa (racemic)** | 7 (0.5%) | 335 (0.4%) | | C02 | Antihypertensives | 7 (100.0%) | 335 (100.0%) | |
| **Metoclopramide** | 122 (8.7%) | 6889 (7.3%) | | A03 | Drugs for functional gastrointestinal disorders | 122 (100.0%) | 6889 (100.0%) | |
| **Metronidazole** | 17 (1.2%) | 889 (0.9%) | | J01 | Antibacterials for systemic use | 10 (55.6%) | 504 (55.0%) | |
|  |  |  |  | G01 | Gynecological antiinfectives | 8 (44.4%) | 413 (45.0%) | |
| **Miconazole** | 134 (9.6%) | 9334 (9.9%) | | G01 | Gynecological antiinfectives | 114 (80.3%) | 6885 (69.4%) | |
|  |  |  |  | D01 | Antifungals for dermatological use | 17 (12.0%) | 2022 (20.4%) | |
|  |  |  |  | A01 | Stomatological preparations | 11 (7.7%) | 1015 (10.2%) | |
| **Minocycline** | 7 (0.5%) | 226 (0.2%) | | J01 | Antibacterials for systemic use | 7 (100.0%) | 226 (100.0%) | |
| **Mometasone** | 31 (2.2%) | 2578 (2.7%) | | D07 | Corticosteroids | 31 (93.9%) | 2578 (93.7%) | |
|  |  |  |  | R01 | Nasal preparations | 2 (6.1%) | 174 (6.3%) | |
| **Monoxerutin** | 21 (1.5%) | 2458 (2.6%) | | C05 | Vasoprotectives | 21 (100.0%) | 2458 (100.0%) | |
| **Morpholine salicylate** | 17 (1.2%) | 1157 (1.2%) | | N02 | Analgesics | 17 (100.0%) | 1157 (100.0%) | |
| **Mupirocin** | 31 (2.2%) | 3484 (3.7%) | | D06 | Antibiotics | 31 (100.0%) | 3345 (95.5%) | |
|  |  |  |  | R01 | Nasal preparations | 0 (0%) | 158 (4.5%) | |
| **Naphazoline** | 18 (1.3%) | 1208 (1.3%) | | R01 | Nasal preparations | 18 (100.0%) | 1208 (100.0%) | |
| **Naproxen** | 23 (1.6%) | 2072 (2.2%) | | M01 | Antiinflammatory | 23 (100.0%) | 2072 (100.0%) | |
| **Nifedipine** | 21 (1.5%) | 1090 (1.2%) | | C08 | Calcium channel blockers | 21 (100.0%) | 1089 (99.9%) | |
|  |  |  |  | C05 | Vasoprotectives | 0 (0%) | 1 (0.1%) | |
| **Nimesulide** | 10 (0.7%) | 357 (0.4%) | | M01 | Antiinflammatory | 10 (100.0%) | 357 (100.0%) | |
| **Norgestimate** | 5 (0.4%) | 303 (0.3%) | | G03 | Sex hormones | 5 (100.0%) | 303 (100.0%) | |
| **Norgestrel** | 7 (0.5%) | 611 (0.6%) | | G03 | Sex hormones | 7 (100.0%) | 611 (100.0%) | |
| **Ofloxacin** | 6 (0.4%) | 265 (0.3%) | | J01 | Antibacterials for systemic use | 6 (100.0%) | 265 (100.0%) | |
| **Omeprazole** | 15 (1.1%) | 972 (1.0%) | | A02 | Drugs for acid related disorders | 15 (100.0%) | 972 (100.0%) | |
| **Orphenadrine** | 6 (0.4%) | 868 (0.9%) | | M03 | Muscle relaxants | 6 (100.0%) | 868 (100.0%) | |
| **Oxolamine** | 28 (2.0%) | 1531 (1.6%) | | R05 | Cough | 28 (100.0%) | 1531 (100.0%) | |
| **Oxomemazine** | 8 (0.6%) | 228 (0.2%) | | R06 | Antihistamines for systemic use | 8 (100.0%) | 228 (100.0%) | |
| **Oxymetazoline** | 21 (1.5%) | 1025 (1.1%) | | R01 | Nasal preparations | 21 (100.0%) | 1025 (100.0%) | |
| **Papaverine** | 85 (6.1%) | 5222 (5.5%) | | A03 | Drugs for functional gastrointestinal disorders | 85 (100.0%) | 5222 (100.0%) | |
| **Phenazopyridine** | 36 (2.6%) | 1716 (1.8%) | | G04 | Urologicals | 36 (100.0%) | 1716 (100.0%) | |
| **Phenoxymethylpenicillin** | 34 (2.4%) | 3329 (3.5%) | | J01 | Antibacterials for systemic use | 34 (100.0%) | 3329 (100.0%) | |
| **Phenylephrine** | 57 (4.1%) | 3193 (3.4%) | | R01 | Nasal preparations | 51 (75.0%) | 2671 (67.5%) | |
|  |  |  |  | R06 | Antihistamines for systemic use | 15 (22.1%) | 1094 (27.7%) | |
|  |  |  |  | D04 | Antipruritics | 2 (2.9%) | 191 (4.8%) | |
| **Phenylephrine comb.** | 68 (4.9%) | 2795 (3.0%) | | R01 | Nasal preparations | 68 (100.0%) | 2795 (100.0%) | |
| **Piroxicam** | 7 (0.5%) | 347 (0.4%) | | M02 | Topical products for joint | 7 (100.0%) | 347 (100.0%) | |
| **Polymyxin B** | 112 (8.0%) | 8546 (9.0%) | | S01 | Ophthalmologicals | 58 (43.0%) | 5273 (51.8%) | |
|  |  |  |  | S02 | Otologicals | 54 (40.0%) | 3353 (33.0%) | |
|  |  |  |  | S03 | Ophthalmological | 14 (10.4%) | 1079 (10.6%) | |
|  |  |  |  | D10 | Anti-acne preparations | 9 (6.7%) | 466 (4.6%) | |
| **Povidone-iodine** | 24 (1.7%) | 2068 (2.2%) | | G01 | Gynecological antiinfectives | 13 (52.0%) | 918 (43.6%) | |
|  |  |  |  | D08 | Antiseptics | 12 (48.0%) | 1121 (53.3%) | |
|  |  |  |  | D09 | Medicated dressings | 0 (0%) | 66 (3.1%) | |
| **Prednisolone** | 17 (1.2%) | 660 (0.7%) | | D07 | Corticosteroids | 17 (100.0%) | 660 (100.0%) | |
| **Prednisone** | 15 (1.1%) | 762 (0.8%) | | H02 | Corticosteroids for systemic use | 15 (100.0%) | 762 (100.0%) | |
| **Progesterone** | 61 (4.4%) | 4552 (4.8%) | | G03 | Sex hormones | 61 (100.0%) | 4552 (100.0%) | |
| **Promethazine** | 12 (0.9%) | 714 (0.8%) | | R06 | Antihistamines for systemic use | 12 (100.0%) | 714 (100.0%) | |
| **Propranolol** | 9 (0.6%) | 254 (0.3%) | | C07 | Beta blocking agents | 9 (100.0%) | 254 (100.0%) | |
| **Pseudoephedrine** | 38 (2.7%) | 1793 (1.9%) | | R01 | Nasal preparations | 38 (100.0%) | 1793 (100.0%) | |
| **Ranitidine** | 17 (1.2%) | 723 (0.8%) | | A02 | Drugs for acid related disorders | 17 (100.0%) | 723 (100.0%) | |
| **Roxithromycin** | 10 (0.7%) | 1103 (1.2%) | | J01 | Antibacterials for systemic use | 10 (100.0%) | 1103 (100.0%) | |
| **Salbutamol** | 34 (2.4%) | 2422 (2.6%) | | R03 | Drugs for obstructive airway diseases | 34 (100.0%) | 2422 (100.0%) | |
| **Salmeterol** | 6 (0.4%) | 320 (0.3%) | | R01 | Nasal preparations | 6 (50.0%) | 196 (38.0%) | |
|  |  |  |  | R03 | Drugs for obstructive airway diseases | 6 (50.0%) | 320 (62.0%) | |
| **Selenium sulfide** | 13 (0.9%) | 649 (0.7%) | | D01 | Antifungals for dermatological use | 13 (100.0%) | 649 (100.0%) | |
| **Senna glycosides** | 13 (0.9%) | 670 (0.7%) | | A06 | Drugs for constipation | 13 (100.0%) | 670 (100.0%) | |
| **Silver sulfadiazine** | 6 (0.4%) | 604 (0.6%) | | D06 | Antibiotics | 6 (100.0%) | 604 (100.0%) | |
| **Sodium chloride** | 23 (1.6%) | 1644 (1.7%) | | R01 | Nasal preparations | 20 (87.0%) | 1418 (85.4%) | |
|  |  |  |  | A12 | Mineral supplements | 3 (13.0%) | 242 (14.6%) | |
| **Sulfacetamide** | 6 (0.4%) | 284 (0.3%) | | S01 | Ophthalmologicals | 6 (100.0%) | 284 (100.0%) | |
| **Sulfamethoxazole** | 11 (0.8%) | 957 (1.0%) | | J01 | Antibacterials for systemic use | 11 (100.0%) | 957 (100.0%) | |
| **Terbinafine** | 5 (0.4%) | 627 (0.7%) | | D01 | Antifungals for dermatological use | 5 (100.0%) | 627 (100.0%) | |
| **Terbutaline** | 7 (0.5%) | 683 (0.7%) | | R03 | Drugs for obstructive airway diseases | 7 (100.0%) | 683 (100.0%) | |
| **Tetracaine** | 14 (1.0%) | 1413 (1.5%) | | S01 | Ophthalmologicals | 14 (100.0%) | 1413 (100.0%) | |
| **Tetryzoline** | 5 (0.4%) | 540 (0.6%) | | S01 | Ophthalmologicals | 5 (71.4%) | 540 (74.1%) | |
|  |  |  |  | R01 | Nasal preparations | 2 (28.6%) | 189 (25.9%) | |
| **Tocopherol (Vit E).** | 46 (3.3%) | 5117 (5.4%) | | A11 | Vitamins | 28 (60.9%) | 2225 (42.6%) | |
|  |  |  |  | B03 | Antianemic preparations | 18 (39.1%) | 2992 (57.4%) | |
| **Tretinoin** | 7 (0.5%) | 373 (0.4%) | | D10 | Anti-acne preparations | 7 (100.0%) | 373 (100.0%) | |
| **Triamcinolone** | 62 (4.4%) | 4667 (4.9%) | | D07 | Corticosteroids | 41 (64.1%) | 3151 (65.8%) | |
|  |  |  |  | A01 | Stomatological preparations | 17 (26.6%) | 1318 (27.5%) | |
|  |  |  |  | R01 | Nasal preparations | 6 (9.4%) | 320 (6.7%) | |
| **Tyrothricin** | 8 (0.6%) | 863 (0.9%) | | R02 | Throat preparations | 8 (100.0%) | 863 (100.0%) | |
| **Various** | 19 (1.4%) | 1346 (1.4%) | | M02 | Topical products for joint | 19 (100.0%) | 1346 (100.0%) | |
| **Vitamin B1 in combination** | 28 (2.0%) | 2655 (2.8%) | | A11 | Vitamins | 28 (100.0%) | 2655 (100.0%) | |
| **Vitamins** | 570 (40.7%) | 40177 (42.4%) | | A11 | Vitamins | 570 (100.0%) | 40177 (100.0%) | |
| **Zinc products** | 13 (0.9%) | 838 (0.9%) | | D02 | Emollients | 13 (100.0%) | 838 (99.6%) | |
|  |  |  |  | D09 | Medicated dressings | 0 (0%) | 3 (0.4%) | |
| **Zinc sulfate** | 7 (0.5%) | 188 (0.2%) | | A12 | Mineral supplements | 7 (100.0%) | 188 (100.0%) | |

The indication columns present the percentage share of indications for each respective drug.

# **Table S3.** Prescription-wide (RxWAS) associations between maternal prescription drug exposure during pregnancy and risk of offspring ASD before and after adjustment for ATC-based indications.

| **Drug prescribed in pregnancy** | **Offspring ASD risk**  **HR [95% CI]** | |
| --- | --- | --- |
|  | **RxWAS Model 1** | **RxWAS Model 2** |
| **Acetylcysteine** | 1.15 [0.68, 1.97] | 1.14 [0.64, 2] |
| **Acetylsalicylic acid** | 0.95 [0.81, 1.1] | 1 [0.8, 1.24] |
| **Aciclovir** | 0.9 [0.53, 1.53] | NA |
| **Alprazolam** | 2.09 [0.92, 4.77] | NA |
| **Amitriptyline** | **3.87 [1.65, 9.04]** | 1.89 [0.74, 4.87] |
| **Amoxicillin** | **0.8 [0.69, 0.93]** | 0.96 [0.78, 1.18] |
| **Anti-D (Rh) immunoglobulin** | 0.77 [0.57, 1.03] | NA |
| **Artificial tears** | 0.55 [0.24, 1.22] | 0.68 [0.3, 1.56] |
| **Azelastine** | 1.68 [0.74, 3.82] | 1.43 [0.62, 3.26] |
| **Azithromycin** | 0.78 [0.41, 1.47] | 0.89 [0.47, 1.69] |
| **Bifonazole** | 0.8 [0.62, 1.02] | 0.9 [0.68, 1.21] |
| **Bismuth preparations** | 0.91 [0.49, 1.72] | 0.87 [0.42, 1.78] |
| **Bromhexine** | 0.86 [0.57, 1.31] | 0.81 [0.52, 1.28] |
| **Bromocriptine** | 1.24 [0.64, 2.42] | NA |
| **Budesonide** | 0.96 [0.57, 1.62] | 1 [0.55, 1.8] |
| **Cabergoline** | 1.01 [0.48, 2.15] | NA |
| **Calcium carbonate** | **2.36 [1.32, 4.24]** | **2.18 [1.17, 4.06]** |
| **Carbamide** | 0.72 [0.35, 1.5] | 0.69 [0.29, 1.61] |
| **Carbocisteine** | 1.06 [0.76, 1.49] | 1.05 [0.71, 1.57] |
| **Cefalexin** | **0.63 [0.44, 0.91]** | 0.72 [0.5, 1.05] |
| Cefuroxime | 1 [0.83, 1.19] | 1.23 [1, 1.51] |
| Celecoxib | 1.21 [0.6, 2.46] | 1.3 [0.63, 2.69] |
| Cetirizine | 1.48 [0.78, 2.77] | 1.27 [0.66, 2.43] |
| Chlorphenamine | 0.86 [0.48, 1.55] | 0.7 [0.38, 1.3] |
| Choriogonadotropin alfa | 0.7 [0.29, 1.72] | 0.74 [0.3, 1.83] |
| **Citalopram** | **2.88 [1.34, 6.2]** | 1.32 [0.55, 3.15] |
| Clarithromycin | 0.69 [0.29, 1.68] | 0.79 [0.33, 1.93] |
| Clindamycin | 1.26 [0.85, 1.86] | 1.21 [0.81, 1.81] |
| Clobetasol | 0.68 [0.39, 1.19] | 0.84 [0.48, 1.49] |
| Clomifene | 1.1 [0.81, 1.48] | 1.24 [0.87, 1.77] |
| Combinations | 1.05 [0.76, 1.44] | 0.85 [0.57, 1.28] |
| Cromoglicic acid | 0.83 [0.33, 2.07] | 1.06 [0.42, 2.7] |
| Cyanocobalamin | 0.92 [0.62, 1.35] | 1.01 [0.68, 1.49] |
| **Cyproterone** | **2.48 [1.09, 5.64]** | **2.71 [1.17, 6.25]** |
| Desloratadine | 1.86 [0.91, 3.78] | 1.61 [0.77, 3.35] |
| Dexamethasone | 0.9 [0.79, 1.04] | 1.09 [0.9, 1.32] |
| Dexpanthenol | 1.08 [0.59, 1.97] | 1.79 [0.6, 5.31] |
| Diazepam | 1.05 [0.47, 2.35] | NA |
| Diclofenac | 0.83 [0.63, 1.1] | 0.9 [0.61, 1.35] |
| Doxycycline | 1.1 [0.7, 1.75] | 1.27 [0.79, 2.02] |
| Drospirenone | 1.92 [0.84, 4.36] | 2.1 [0.91, 4.84] |
| Enoxaparin | 0.96 [0.55, 1.66] | NA |
| Erythromycin | 1.02 [0.69, 1.5] | 1.01 [0.68, 1.51] |
| Estradiol | 1.03 [0.64, 1.66] | 1.11 [0.67, 1.83] |
| Etodolac | 0.78 [0.46, 1.32] | 0.8 [0.46, 1.4] |
| Famotidine | 1.21 [0.8, 1.82] | 1.04 [0.65, 1.68] |
| **Ferrous sulfate** | **0.77 [0.68, 0.87]** | **0.82 [0.68, 0.99]** |
| Fexofenadine | 0.93 [0.59, 1.47] | 0.77 [0.47, 1.25] |
| Fluconazole | 1.75 [1, 3.07] | NA |
| **Fluoxetine** | **3.94 [1.69, 9.21]** | 1.88 [0.73, 4.83] |
| Fluticasone | 1.29 [0.95, 1.76] | 1.14 [0.82, 1.6] |
| **Folic acid** | 1.11 [0.97, 1.28] | **1.47 [1.23, 1.76]** |
| Fosfomycin | 0.88 [0.46, 1.69] | 1.01 [0.52, 1.93] |
| Fusidic acid | 1.11 [0.46, 2.69] | 1.43 [0.57, 3.54] |
| **Ganirelix** | **2.64 [1.05, 6.67]** | NA |
| Gentamicin | 0.81 [0.43, 1.51] | 1.04 [0.54, 2.01] |
| Gestodene | 1.13 [0.71, 1.82] | 1.23 [0.75, 2.02] |
| Glycerol | 0.63 [0.33, 1.22] | NA |
| Hepatitis B | 1.62 [0.88, 2.97] | NA |
| Ipratropium bromide | 0.61 [0.21, 1.73] | 0.61 [0.21, 1.77] |
| Isoconazole | 0.72 [0.52, 1.02] | 0.92 [0.64, 1.34] |
| Ispaghula | 1.43 [0.62, 3.31] | NA |
| Ispaghula (psylla seeds) | 1.27 [0.46, 3.48] | NA |
| Ketoconazole | 0.91 [0.55, 1.5] | NA |
| Lactulose | 1.27 [0.66, 2.46] | NA |
| Levocabastine | 1.76 [0.81, 3.84] | 2.09 [0.94, 4.64] |
| Levothyroxine sodium | 1.13 [0.8, 1.59] | NA |
| Lidocaine | 0.71 [0.36, 1.37] | 0.75 [0.36, 1.57] |
| Loperamide | 0.97 [0.59, 1.6] | 0.96 [0.48, 1.93] |
| **Loratadine** | **1.41 [1.03, 1.93]** | 1.3 [0.89, 1.88] |
| Lorazepam | 1.57 [0.73, 3.35] | NA |
| **Lynestrenol** | **0.42 [0.2, 0.9]** | **0.43 [0.2, 0.93]** |
| Mebendazole | 0.89 [0.47, 1.72] | NA |
| **Medicinal charcoal** | **2.93 [1.06, 8.09]** | **3.33 [1.13, 9.82]** |
| Metamizole sodium | 0.78 [0.53, 1.15] | 0.85 [0.56, 1.29] |
| Metformin | 1.58 [0.72, 3.48] | NA |
| Methyldopa (racemic) | 1.21 [0.57, 2.57] | NA |
| Metoclopramide | 1.16 [0.95, 1.43] | NA |
| Metronidazole | 1.31 [0.78, 2.2] | 1.39 [0.82, 2.36] |
| Miconazole | 0.99 [0.81, 1.19] | 1.02 [0.78, 1.32] |
| Minocycline | 1.51 [0.71, 3.23] | 1.74 [0.81, 3.73] |
| Mometasone | 0.79 [0.54, 1.15] | 0.96 [0.65, 1.44] |
| Monoxerutin | 0.69 [0.44, 1.1] | 0.69 [0.37, 1.29] |
| Morpholine salicylate | 0.97 [0.58, 1.61] | 1.09 [0.64, 1.85] |
| Mupirocin | 0.69 [0.47, 1.01] | 0.71 [0.47, 1.07] |
| Naphazoline | 0.93 [0.58, 1.49] | 0.77 [0.47, 1.25] |
| Naproxen | 0.8 [0.5, 1.27] | 0.8 [0.48, 1.34] |
| Nifedipine | 1.26 [0.76, 2.11] | NA |
| Nimesulide | 1.54 [0.81, 2.91] | 1.68 [0.86, 3.28] |
| Norgestimate | 1.17 [0.47, 2.93] | 1.26 [0.49, 3.21] |
| Norgestrel | 0.75 [0.35, 1.59] | 0.8 [0.37, 1.71] |
| Ofloxacin | 2.12 [0.85, 5.29] | 2.41 [0.96, 6.02] |
| Omeprazole | 0.95 [0.56, 1.6] | 0.79 [0.45, 1.39] |
| **Orphenadrine** | **0.42 [0.19, 0.95]** | NA |
| Oxolamine | 1.11 [0.76, 1.63] | 1.1 [0.72, 1.69] |
| **Oxomemazine** | **2.21 [1.07, 4.58]** | 1.92 [0.91, 4.07] |
| Oxymetazoline | 1.39 [0.87, 2.21] | 1.18 [0.72, 1.91] |
| Papaverine | 1.07 [0.84, 1.36] | NA |
| Phenazopyridine | 1.35 [0.94, 1.92] | NA |
| Phenoxymethylpenicillin | 0.77 [0.55, 1.09] | 0.9 [0.63, 1.28] |
| Phenylephrine | 1.21 [0.92, 1.59] | 1.01 [0.74, 1.37] |
| **Phenylephrine comb.** | **1.52 [1.17, 1.97]** | 1.34 [0.99, 1.79] |
| Piroxicam | 1.38 [0.65, 2.96] | NA |
| Polymyxin B | 0.88 [0.71, 1.08] | 0.96 [0.69, 1.34] |
| Povidone-iodine | 0.71 [0.47, 1.07] | 0.69 [0.41, 1.17] |
| **Prednisolone** | **1.65 [1.01, 2.69]** | **2.1 [1.27, 3.49]** |
| Prednisone | 1.18 [0.7, 1.99] | 1.3 [0.62, 2.75] |
| Progesterone | 0.82 [0.62, 1.1] | 0.83 [0.59, 1.17] |
| Promethazine | 1.22 [0.65, 2.28] | 1.03 [0.54, 1.98] |
| **Propranolol** | **2.11 [1.08, 4.16]** | NA |
| **Pseudoephedrine** | **1.47 [1.04, 2.08]** | 1.27 [0.88, 1.84] |
| Ranitidine | 1.4 [0.86, 2.27] | 1.23 [0.72, 2.1] |
| Roxithromycin | 0.53 [0.29, 1] | 0.61 [0.32, 1.14] |
| Salbutamol | 0.87 [0.61, 1.24] | 0.83 [0.51, 1.36] |
| Salmeterol | 1.17 [0.46, 2.99] | 1.12 [0.42, 2.94] |
| Selenium sulfide | 1.23 [0.68, 2.22] | 1.42 [0.78, 2.59] |
| Senna glycosides | 1.62 [0.8, 3.26] | NA |
| Silver sulfadiazine | 0.69 [0.31, 1.55] | 0.75 [0.33, 1.7] |
| Sodium chloride | 0.98 [0.63, 1.53] | NA |
| Sulfacetamide | 1.4 [0.62, 3.17] | 1.82 [0.79, 4.22] |
| Sulfamethoxazole | 1.02 [0.45, 2.29] | 1.18 [0.52, 2.67] |
| Terbinafine | 0.9 [0.28, 2.92] | 1.03 [0.31, 3.38] |
| Terbutaline | 0.81 [0.35, 1.87] | 0.82 [0.34, 1.96] |
| Tetracaine | 0.93 [0.47, 1.8] | 1.21 [0.61, 2.41] |
| Tetryzoline | 0.62 [0.26, 1.5] | 0.73 [0.3, 1.81] |
| **Tocopherol (Vit E).** | **0.7 [0.51, 0.97]** | 0.76 [0.55, 1.05] |
| Tretinoin | 1.28 [0.55, 2.98] | 1.15 [0.49, 2.72] |
| Triamcinolone | 0.87 [0.66, 1.14] | 1.07 [0.78, 1.46] |
| Tyrothricin | 0.61 [0.29, 1.25] | 0.33 [0.1, 1.06] |
| Various | 0.97 [0.6, 1.59] | NA |
| Vitamin B1 in combination | 0.69 [0.46, 1.02] | NA |
| Vitamins | 0.89 [0.78, 1] | NA |
| Zinc products | 0.87 [0.5, 1.51] | NA |
| **Zinc sulfate** | **2.4 [1.11, 5.15]** | NA |

Model 1 adjusts for maternal age at delivery, number of encounters with healthcare services, and child sex and year of birth; Model 2 adjusts for covariates in Model 1 and ATC-based indications

# **Table S4.** Associations between maternal prescription drug exposure and risk of offspring ASD by pharmacologic target.

| **Pharmacologic target** | **Drug prescribed in pregnancy** | **Target analysis Model 1** | **Target analysis Model 2** |
| --- | --- | --- | --- |
|  |  | **Offspring ASD risk HR [95% CI]** | |
| Acetylcholine receptor; alpha1/beta1/delta/gamma | Oxomemazine● | 0.99 [0.62, 1.6] | 0.96 [0.58, 1.59] |
|  | Lynestrenol●◊ |  |  |
|  | Medicinal charcoal●◊ |  |  |
| ADAM17 | Sulfacetamide | 1.1 [0.72, 1.67] | 1.41 [0.91, 2.18] |
|  | Prednisolone●◊ |  |  |
| Beta-2 adrenergic receptor | Salbutamol | 0.99 [0.72, 1.36] | 1 [0.62, 1.59] |
|  | Propranolol●- |  |  |
| **Butyrylcholinesterase** | Oxomemazine● | **2.18 [1.23, 3.86]** | **2.04 [1.13, 3.69]** |
|  | Cyproterone●◊ |  |  |
| **Carbonic anhydrase IX** | Cyproterone●◊ | 1.67 [0.99, 2.81] | **1.81 [1.05, 3.13]** |
|  | Celecoxib |  |  |
| Cytochrome P450 2D6 | Miconazole | 1 [0.88, 1.13] | 1.03 [0.86, 1.23] |
|  | Dexamethasone |  |  |
|  | Loratadine● |  |  |
|  | Phenylephrine |  |  |
|  | Oxymetazoline |  |  |
|  | Metoclopramide- |  |  |
|  | Bromhexine |  |  |
|  | Fluoxetine● |  |  |
|  | Orphenadrine●- |  |  |
|  | Azelastine |  |  |
|  | Celecoxib |  |  |
|  | Terbinafine |  |  |
| G-protein coupled bile acid receptor 1 | Sulfacetamide | 1.1 [0.72, 1.67] | 1.41 [0.91, 2.18] |
|  | Prednisolone●◊ |  |  |
| Glucocorticoid receptor | Dexamethasone | 0.96 [0.83, 1.11] | 0.99 [0.82, 1.2] |
|  | Clobetasol |  |  |
|  | Fluticasone |  |  |
|  | Progesterone |  |  |
|  | Lidocaine |  |  |
|  | Polymyxin b |  |  |
|  | Triamcinolone |  |  |
|  | Salmeterol |  |  |
|  | Budesonide |  |  |
|  | Sulfacetamide |  |  |
|  | Prednisolone●◊ |  |  |
| Interleukin-6 | Sulfacetamide | 1.1 [0.72, 1.67] | 1.41 [0.91, 2.18] |
|  | Prednisolone●◊ |  |  |
| Mineralocorticoid receptor | Dexamethasone | 1.05 [0.9, 1.23] | 1.04 [0.85, 1.28] |
|  | Fluticasone |  |  |
|  | Progesterone |  |  |
|  | Polymyxin b |  |  |
|  | Salmeterol |  |  |
|  | Budesonide |  |  |
|  | Sulfacetamide |  |  |
|  | Prednisolone●◊ |  |  |
| Muscarinic acetylcholine receptor M1 | Acetylsalicylic acid | 1.01 [0.69, 1.47] | 0.97 [0.6, 1.56] |
|  | Orphenadrine●- |  |  |
|  | Desloratadine |  |  |
|  | Promethazine |  |  |
|  | Amitriptyline● |  |  |
| Muscarinic acetylcholine receptor M2 | Orphenadrine●- | 0.99 [0.67, 1.47] | 0.96 [0.58, 1.59] |
|  | Desloratadine |  |  |
|  | Promethazine |  |  |
|  | Amitriptyline● |  |  |
| Muscarinic acetylcholine receptor M3 | Acetylsalicylic acid | 1.03 [0.72, 1.47] | 0.97 [0.61, 1.55] |
|  | Orphenadrine●- |  |  |
|  | Desloratadine |  |  |
|  | Promethazine |  |  |
|  | Amitriptyline● |  |  |
| Muscarinic acetylcholine receptor M4 | Acetylsalicylic acid | 1.01 [0.69, 1.47] | 0.96 [0.59, 1.54] |
|  | Orphenadrine●- |  |  |
|  | Desloratadine |  |  |
|  | Promethazine |  |  |
|  | Amitriptyline● |  |  |
| Muscarinic acetylcholine receptor M5 | Acetylsalicylic acid | 0.99 [0.69, 1.42] | 0.9 [0.57, 1.43] |
|  | Orphenadrine●- |  |  |
|  | Desloratadine |  |  |
|  | Promethazine |  |  |
|  | Amitriptyline● |  |  |
| **Neuronal acetylcholine receptor; alpha4/beta4** | Pseudoephedrine● | **1.64 [1.22, 2.21]** | **1.45 [1.05, 1.99]** |
|  | Oxomemazine● |  |  |
|  | Cyproterone●◊ |  |  |
| Peptidyl-prolyl cis-trans isomerase NIMA-interacting 1 | Cyproterone●◊ | 0.86 [0.53, 1.39] | 0.86 [0.53, 1.42] |
|  | Lynestrenol●◊ |  |  |
|  | Tretinoin |  |  |
| Serotonin 2a (5-HT2a) receptor | Acetylsalicylic acid | 1.05 [0.78, 1.42] | 0.96 [0.66, 1.39] |
|  | Fluoxetine● |  |  |
|  | Orphenadrine●- |  |  |
|  | Desloratadine |  |  |
|  | Bromocriptine- |  |  |
|  | Promethazine |  |  |
|  | Amitriptyline● |  |  |
| **Serotonin 2b (5-HT2b) receptor** | Acetylsalicylic acid | **1.31 [1.11, 1.55]** | **1.31 [1.04, 1.65]** |
|  | Loratadine● |  |  |
|  | Metoclopramide- |  |  |
|  | Desloratadine |  |  |
|  | Propranolol●- |  |  |
|  | Bromocriptine- |  |  |
|  | Promethazine |  |  |
|  | Amitriptyline● |  |  |
|  | Tretinoin |  |  |
| Serotonin 2c (5-HT2c) receptor | Acetylsalicylic acid | 1.22 [0.96, 1.54] | 1.09 [0.83, 1.45] |
|  | Phenylephrine |  |  |
|  | Oxymetazoline |  |  |
|  | Fluoxetine● |  |  |
|  | Orphenadrine●- |  |  |
|  | Citalopram● |  |  |
|  | Desloratadine |  |  |
|  | Bromocriptine- |  |  |
|  | Promethazine |  |  |
|  | Amitriptyline● |  |  |
| Serotonin 3a (5-HT3a) receptor | Lynestrenol●◊ | 1.08 [0.7, 1.65] | 1.13 [0.72, 1.77] |
|  | Medicinal charcoal●◊ |  |  |
|  | Calcium carbonate●◊ |  |  |
| Serotonin transporter | Miconazole | 1.01 [0.85, 1.19] | 0.99 [0.8, 1.23] |
|  | Ketoconazole- |  |  |
|  | Gestodene |  |  |
|  | Fluoxetine● |  |  |
|  | Orphenadrine●- |  |  |
|  | Citalopram● |  |  |
|  | Desloratadine |  |  |
|  | Propranolol●- |  |  |
|  | Amitriptyline● |  |  |

● = associated with ASD in RxWAS Model 1; ◊ = associated with ASD in RxWAS Model 2; - = could not be included in Model 2; Model 1 adjusts for maternal age at delivery, number of encounters with healthcare services, and child sex and year of birth; Model 2 adjusts for covariates in Model 1 and ATC-based indications

# **Figure S1.** Target-based analysis flow.


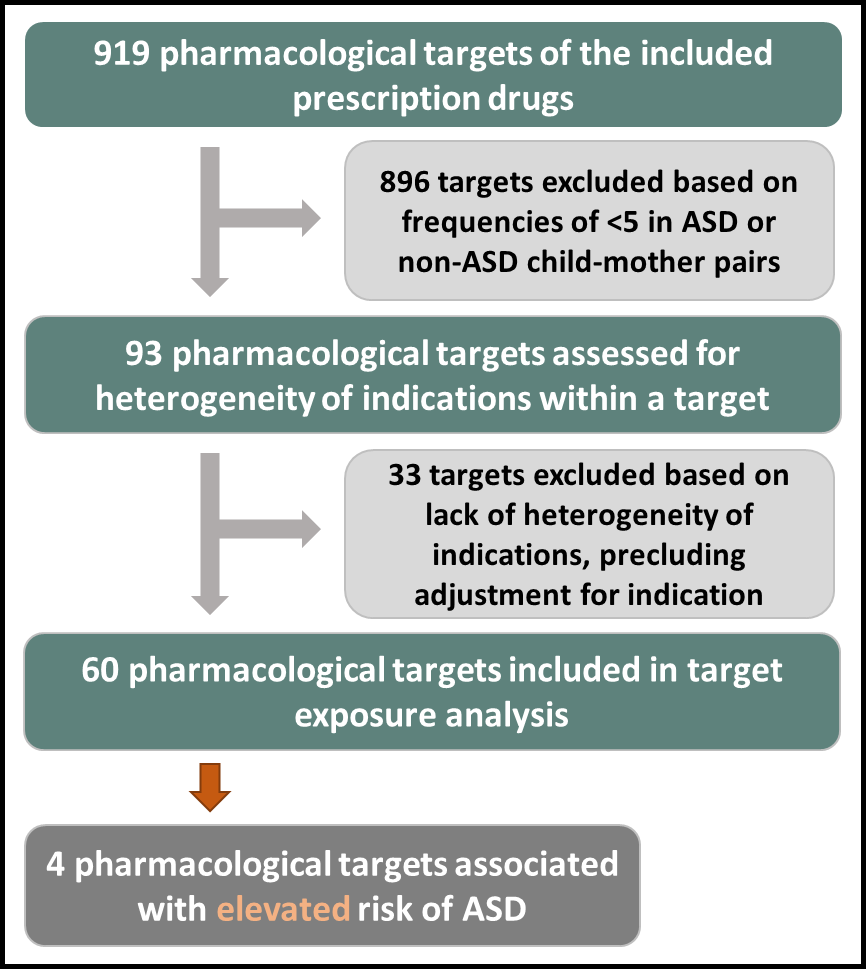


**REFERENCES**

1. Janecka, M., et al., *Association of Autism Spectrum Disorder With Prenatal Exposure to Medication Affecting Neurotransmitter Systems.* JAMA Psychiatry, 2018. **75**(12): p. 1217-1224.

2. Levine, S.Z., et al., *Association of Maternal Use of Folic Acid and Multivitamin Supplements in the Periods Before and During Pregnancy With the Risk of Autism Spectrum Disorder in Offspring.* JAMA Psychiatry, 2018. **75**(2): p. 176-184.

3. Davidovitch, M., et al., *Prevalence and incidence of autism spectrum disorder in an Israeli population.* J Autism Dev Disord, 2013. **43**(4): p. 785-93.

4. Reilly, M., *Controlled epidemiological studies*. First edition. ed. Chapman & Hall/CRC Press biostatistics series. 2023, Boca Raton: CRC Press.

5. Greenland, S., M.A. Mansournia, and D.G. Altman, *Sparse data bias: a problem hiding in plain sight.* BMJ, 2016. **352**: p. i1981.
